# Genetic and molecular mechanisms of the FOXP2-CNTNAP2 pathway in Autism spectrum disorders and speech disorders

**DOI:** 10.1007/s00702-026-03160-w

**Published:** 2026-07-15

**Authors:** Fanglin Song, Xin Li, Cheng Cheng, Jiacheng Du

**Affiliations:** 1 School of Medicine, Orthopaedics, Trauma and Sports Medicine, University of Nottingham, Queen’s Medical Centre, F Floor West Block, Nottingham, NG7 2RD UK; 2 Institute of Division of Surgery and Interventional Science, University College London, University College London, Gower Street, London, WC1E 6BT UK; 3 School of Humanities, Beijing University of Chinese Medicine, No. 11, North 3rd Ring East Road, Chaoyang District, Beijing, 100029 China

**Keywords:** ASD, speech disorder, FOXP2, CNTNAP2, pathway

## Abstract

Autism spectrum disorders (ASD) and speech disorders are neurodevelopmental disorders whose etiology involves intricate genetic and molecular mechanisms. In recent years, the FOXP2-CNTNAP2 pathway has been identified as playing a crucial role in language development and neural function. Aberrant expression or mutation within this pathway is closely associated with the pathogenesis of ASD and speech disorders. Nevertheless, the specific regulatory mechanisms of this pathway and its pathological role in these diseases have not been fully clarified. Therefore, a systematic review of existing research is urgently required to elucidate its molecular underpinnings. An in-depth analysis of the genetic and molecular mechanisms of the FOXP2-CNTNAP2 pathway will not only contribute to understanding the pathogenesis of ASD and speech disorders but also offer potential molecular markers for diagnosing related conditions. Moreover, it provides a theoretical foundation for developing targeted therapeutic strategies. Additionally, this research may offer a novel perspective for studying genetic regulatory networks in neurodevelopment, holding significant scientific value and clinical translation potential.

## Introduction

Autism Spectrum Disorder (ASD) and speech disorders are complex neurodevelopmental disorders whose etiology involves the interaction of genetic, molecular, and environmental factors (Hodges et al. 2020). In recent years, the FOXP2-CNTNAP2 pathway has attracted much attention due to its critical role in developing language-related neural circuits. Studies have shown that the transcription factor FOXP2 affects neuronal migration, synapse formation, and axonal myelination by regulating the expression of its target gene CNTNAP2. Abnormalities in this pathway are closely associated with language deficits and speech disorders in ASD patients (Fisher and Scharff 2009). Genome-wide association analysis (GWAS) and animal model studies further support the central role of the FOXP2-CNTNAP2 pathway in language and social behavior (SaravananKA, Panigrahi et al. 2023). However, its specific molecular mechanisms and synergistic effects with other ASD risk genes still require in-depth exploration.

Studying this topic is of great significance. On the one hand, understanding the FOXP2-CNTNAP2 pathway may provide a potential biomarker for the early diagnosis of ASD and speech disorders. On the other hand, by unraveling the regulatory mechanism of this pathway, it may provide a theoretical basis for developing targeted intervention strategies (e.g., gene therapy or pharmacological modulation). Furthermore, studying this pathway contributes to understanding the evolutionary and genetic basis of human language ability. This review aims to systematically outline the genetic basis and molecular mechanisms of the FOXP2-CNTNAP2 pathway in ASD and speech disorders. By integrating existing evidence, we hope to provide a theoretical framework for further research in this field and promote the progress of related clinical applications.

## ASD and speech disorders

Autism Spectrum Disorder (ASD) is frequently associated with distinct cerebellar abnormalities, which may contribute to the language and motor impairments observed in affected individuals. Neuroimaging and postmortem studies have consistently reported cerebellar hypoplasia, particularly in the vermis and hemispheres, along with reduced Purkinje cell density and altered synaptic morphology (Varghese et al. 2017, Brignell et al. 2018, Ferland et al. 2003). These structural alterations are paralleled by functional deficits, including disrupted cerebello-thalamo-cortical connectivity and impaired sensorimotor integration (Fujita et al. 2012, Fujita et al. 2008). Given the cerebellum’s established roles in motor coordination, timing, and procedural learning—processes essential for fluent speech and prosody—its dysfunction in ASD provides a plausible neurobiological substrate for the overlapping language and communication deficits characteristic of the disorder.

There is a strong association between ASD and speech disorders, but the relationship is complex and diverse. Approximately 64% of children with ASD also have a speech delay, while 37% of those with a speech delay also have ASD (Brignell et al. 2018). This suggests a high co-morbidity rate between the two conditions. Meanwhile, cerebellar damage has been found to be associated with speech delay. In particular, damage to the cerebellar vermis may lead to symptoms similar to those of ASD, potentially explaining the physiological reasons for speech problems in some ASD patients (Varghese et al. 2017). Moreover, delayed speech and motor development have been observed in several cases of ASD patients (Thomas et al. 2018), further supporting the association between ASD and speech/motor function.

Language impairments in ASD encompass phonological processing deficits (e.g., atypical prosody, intonation abnormalities) and motor speech disorders (e.g., childhood apraxia of speech, oral sensorimotor deficits). Phonological and rhythmic abnormalities and language processing deficits are present in patients with ASD. Children with ASD exhibit atypical rhythmic features in natural social interactions, including abnormalities in intonation modulation, voice quality, and rhythm. These features correlate with symptom severity, especially in patients with better speech and language skills who may still exhibit these problems (Godel et al. 2023). Machine learning-based speech analysis reveals that individuals with ASD exhibit distinct intonation, rhythm, and other prosodic features compared to typically developing populations. These differences can serve as early screening markers. Moreover, targeted interventions, such as intonation-enhancing therapies, have effectively improved communication skills in this population (Asghari et al. 2021). The development of a customized rhyming trait detection model with an accuracy of 91.25% in differentiating children with ASD further validates the objective presence of rhyming disorders, indicating that rhyme is an important diagnostic indicator (Righi et al. 2018).Individuals with ASD have an advantage in pitch perception, but this can also lead to problems in language development, especially in rhyme-related aspects. Although they may have pitch sensitivity, excessive attention to pitch details may interfere with processing the overall rhythm of language (e.g., emotional intonation). The processing of emotion or intonation may be disjointed, which may affect rhythm (Hisaizumi and Tantam 2024). Individuals with high-functioning autism (HFA) and Asperger’s syndrome (AS) have more misarticulation, inappropriate intonation, and resonance problems in their rhythmic and phonological features than typically developing children, which may affect communication (Filipe et al. 2014).

Neuroimaging studies have provided a biological basis for rhythmic disorders. It has been found that the function of the serotonin system in the right insula of patients with ASD correlates with speech ability and that this brain region is involved in emotional intonation processing (Yoshimura et al. 2020). Abnormalities in structural and functional connectivity in language-related brain regions (e.g., Broca’s area, Wernicke’s area) in patients with ASD may lead to difficulties in rhythmic control (Verly et al. 2014). This suggests that even ASD patients with better language skills may still be impaired in speech rhythms due to neurodevelopmental differences, perceptual preferences, and higher-order integration deficits. These impairments are underpinned by abnormalities in language-related brain regions (Broca’s area, Wernicke’s area) and motor coordination circuits (cerebellum, basal ganglia), reflecting a convergence of perceptual, motor, and integrative dysfunctions.

Children with ASD have deficits in nonverbal working memory and morphosyntactic processing that affect language comprehension and expression (Ellis Weismer et al. 2017). In addition, the development of higher-level speech (e.g., metaphor comprehension) is limited by deficits at the phonological, social, and lexico-grammatical levels (Carriedo et al. 2016). Studies have shown significant heterogeneity in the language abilities of children with ASD, with some having normal language and others significantly below age expectations. Children with ASD who have impaired language behave similarly to those with specific language impairment (SLI), suggesting that there may be overlapping mechanisms between the two (Kjelgaard and Tager-Flusberg 2001). Individuals with ASD often have delayed motor development, and some children with ASD suffer from apraxia, an impaired motor program of the muscles required for articulation, which leads to difficulties in sound production (Shriberg et al. 2011).

ASD is a communication and behavioral disorder that affects social interaction, speech development, and typical behavioral development. The core symptoms of ASD (social deficits) and language problems exacerbate each other. For example, a lack of social interaction may limit opportunities for language input, and language deficits further impede social communication (Tsai et al. 2020). A hallmark symptom of ASD is communication difficulties, including speech and language delays (Adaralegbe et al. 2022), which suggests that speech disorders are common in ASD. Individuals with ASD have difficulties with language comprehension, speech, and nonverbal communication. They also exhibit patterns of faulty language use, such as repetitive or rigid language, narrow interests, and uneven language development, which may lead to impaired social interactions (Russo 2014). Children with ASD have a weaker neural response to speech and are 70% more inclined to listen to computer-generated sounds rather than human speech, which may lead to language learning difficulties (Wang et al. 2018).

Neuroimaging studies have shown that children with ASD have significantly lower activation in brain regions related to speech processing than typically developing children (Wang et al. 2025). The language abilities of individuals with ASD vary greatly. Some may have no language at all, while others exhibit repetitive speech (e.g., parroting), abnormal intonation (e.g., mechanical sounds or high-pitched tones), or uneven language development (e.g., demonstrating exceptional competence in specific domains). Studies have shown that in the case of children with ASD, there is a 94% probability of being diagnosed with ASD if their attention to soft speech is less than 30% (Wang et al. 2018). Additionally, speech disorders in children with ASD may be associated with oral motor disorders involving articulation errors, phonological errors, and motor speech disorders (e.g., childhood apraxia of speech) (Broome et al. 2017). Children with ASD have oral sensorimotor deficits that lead to speech and feeding problems. They may have articulation errors, incomplete motor planning, and poor neuromuscular coordination. These are mainly attributed to localized oral sensorimotor deficits, whereas severe problems such as dysarthria may be related to central deficits (Chaware et al. 2021). Chewing and swallowing problems are present in children with autism. They may tear food with their hands, have oral sensory hypersensitivity leading to stuffing, and prefer soft foods. These may be associated with oral motor deficits, such as inefficient chewing, but studies have also mentioned the absence of significant swallowing difficulties, which may indicate that the problems are mainly focused on oral sensory rather than motor aspects (Tkachuk et al. 2021). Some children may present with non-functional oral movements, such as tongue biting, tongue protrusion, or unconscious jaw movements, which are similar to tardive dyskinesia but have a different etiology (the latter is more often caused by long-term substance use) (Hauser et al. 2022). Taken together, oral motor deficits in ASD mainly involve sensory-motor integration problems that affect speech and feeding. A distinction needs to be made between disorders caused by central and local factors.

Language impairment in ASD results from the interaction of social cognitive deficits, neurodevelopmental anomalies, and the environment. The nature of ASD differs from that of a simple language disorder, as it includes not only abnormalities in language structure (e.g., syntax and semantics) but also, more fundamentally, difficulties in pragmatics and social communication. Early identification must be combined with a comprehensive assessment of social behavior and language development, and multifaceted interventions are needed to improve communication function and social integration.

### FOXP2 ‘language gene’

The discovery of the FOXP2 gene stems from the study of the KE family in the UK. Nearly half of the three generations of this family suffer from inherited speech disorders, manifested as severe speech apraxia (e.g., dysphagia and grammatical defects). Genetic analysis revealed that affected members have mutations in the FOXP2 gene, marking the first time a gene was directly linked to speech disorders (Fisher et al. 1998). Brain imaging shows that patients with FOXP2 gene mutations have structural and functional abnormalities in the basal ganglia and frontal cortex, which affect oral motor control and language processing (Lai et al. 2001). As a transcription factor, FOXP2 is involved in brain development. It particularly affects the neural structures related to motor skills and language and influences the mechanism of language disorders by regulating other genes, such as CNTNAP2 (Premi et al. 2012). In neural development, FOXP2 regulates neurite growth and neuronal connection formation. It also regulates genes involved in neurite growth, including those mediating synapse formation and axon guidance. miRNAs have been found to affect neuronal migration by regulating FOXP2 expression (Vernes et al. 2011). These mechanisms may be related to forming language-related neural circuits. FOXP2 influences the cerebral cortical-basal ganglia connection by regulating synaptic plasticity and neuronal migration, which is crucial for language motor control (e.g., pronunciation and grammar processing) (Enard et al. 2009). FOXP2 regulates cortical neurogenesis, promotes the transformation of radial glial cells into intermediate progenitors, and affects neuronal generation. Overexpression or mutation of FOXP2 may change the efficiency of neurogenesis, suggesting its key role in cortical development (Tsui et al. 2013).

FOXP2 exhibits a conserved expression pattern in brain regions critical for vocal learning and motor control, including the basal ganglia (especially the striatum), cerebellar Purkinje cells, and cortical layers (Takahashi et al. 2003). Its expression is dynamically regulated during behavior—such as acute downregulation during song practice in zebra finches—suggesting a role in neuroplasticity and adaptive vocal learning. In mouse models, disruption of Foxp2 impairs ultrasonic vocalization, particularly during pup-mother separation, underscoring the gene’s role in vocal and motor coordination. Specifically, biallelic knockout of Foxp2 leads to dyskinesia, early lethality, and loss of ultrasonic vocalization upon separation, whereas heterozygous deletion results in abnormal vocalization patterns (Shu et al. 2005). This suggests that FOXP2 regulates vocalization in a dose-dependent manner. Transgenic mouse experiments have shown that human FOXP2 can enhance synaptic plasticity, while the mutant gene causes motor sequence learning difficulties (Zucker et al. 2010).

Furthermore, studies on zebra finches have shown that FOXP2 knockdown reduces the dendritic spine density of basal ganglia neurons and damages synaptic plasticity, while miRNAs (such as miR-9 and miR-132) regulate neuronal migration by inhibiting FOXP2 expression (Clovis et al. 2012). FOXP2 also plays a key role in songbird song learning. In songbirds such as zebra finches, FOXP2 is highly expressed in Area X of the basal ganglia nucleus, which is crucial for song learning (Teramitsu et al. 2010). When FOXP2 levels are down-regulated, it can lead to inaccurate song imitation in songbirds, similar to the situation in human speech disorders (Haesler et al. 2007). Dynamic regulation of FOXP2 is essential for song learning, not just at the absolute level, suggesting that FOXP2 may play a role in neuroplasticity (Heston and White 2015). Acute downregulation of FOXP2, such as during singing practice, is associated with increased vocal variability, possibly promoting song plasticity by regulating downstream genes (Miller et al. 2010). Additionally, social situations (e.g., solitary versus courtship tweets) influence FOXP2 regulation patterns, suggesting a neural mechanism involved in behavioral adaptation (Chen and Hong 2018). Adult zebra finches have sharply down-regulated FOXP2 expression during singing, especially when singing alone. This down-regulation is associated with vocal variability and may facilitate song adjustment and learning (Miller et al. 2008). This suggests that FOXP2 plays a role not only in development but also in regulating behavior in adulthood.

FOXP2 dysfunction is consistently linked to cerebellar pathology, particularly in Purkinje cells(Fujita et al. 2008) FOXP2 exhibits a distinct spatiotemporal expression pattern within the developing and mature cerebellum. During embryonic and early postnatal stages in mice, FOXP2 is robustly expressed in cerebellar Purkinje cells, the sole output neurons of the cerebellar cortex, and in the deep cerebellar nuclei (Ferland et al. 2003, Fujita et al. 2008). This developmental expression is crucial for Purkinje cell dendritogenesis, synaptic protein expression (e.g., synaptophysin), and the establishment of proper cerebellar circuit integrity (Fujita et al. 2008, Schulz et al. 2010, Groszer et al. 2008). The functional significance of FOXP2 in the cerebellum thus has a strong developmental component, as evidenced by structural and functional cerebellar deficits in Foxp2 mutant mice, including Purkinje cell dendritic atrophy, reduced spine density, impaired synaptic plasticity, and altered excitability—abnormalities also commonly observed in postmortem and neuroimaging studies of individuals with ASD (Fujita et al. 2012, Khouri-Farah et al. 2024). These cellular and synaptic deficits contribute to motor coordination and vocalization impairments, highlighting the role of FOXP2 in cerebellar circuit integrity.

In addition to its developmental role, emerging evidence suggests FOXP2 continues to be expressed in the adult cerebellum and may contribute to ongoing cerebellar function. In adult mice and songbirds, FOXP2 expression persists in Purkinje cells, albeit at potentially modulated levels (Takahashi et al. 2003, Ferland et al. 2003). While its primary impact on cerebellar structure is established during development, this sustained expression hints at a role in adult cerebellar plasticity, potentially related to motor learning, timing, and the fine-tuning of vocal-motor coordination—functions critical for speech and prosody (Heston and White 2015, Miller et al. 2008). Therefore, the functional significance of cerebellar FOXP2 is likely dual, involving essential roles in circuit formation during development and contributions to the plasticity and maintenance of motor and timing functions in adulthood.

These structural deficits contribute to motor coordination and vocalization impairments, highlighting the role of FOXP2 in cerebellar circuit integrity. The highly expressed pattern of FOXP2 in the basal ganglia and cortex is highly conserved in humans, mice, and songbirds, but its specific role in language-related functions may vary by species (Li et al. 2007).

The accelerated evolution of FOXP2 in echolocating bats may be related to sensorimotor coordination, expanding the functional diversity of FOXP2 in different species (Li et al. 2007). Comparing the evolution of FOXP2 across different species revealed that although the gene is involved in vocalization learning in both humans and songbirds, the specific amino acid replacements are not the same, suggesting that there may be different evolutionary pathways (Webb and Zhang 2005).

Notably, FOXP2 underwent human-specific evolutionary changes not observed in other primates, which may have contributed to the emergence of advanced language capabilities in humans. Comparative genomic studies have identified two amino acid substitutions (Thr303Asn and Asn325Ser) in the human FOXP2 protein that arose after the human lineage diverged from chimpanzees (Enard et al. 2002, Zhang et al. 2002). These changes are thought to influence FOXP2’s function as a transcription factor, potentially altering its DNA-binding properties or interaction with co-regulators, thereby affecting the expression of downstream genes involved in neural circuit development and plasticity (Zhang et al. 2002, Konopka et al. 2009). The human-specific FOXP2 variant, when expressed in mice, has enhanced synaptic plasticity and facilitated more rapid motor-skill learning (Enard et al. 2009), suggesting that these evolutionary modifications may have fine-tuned neural circuits essential for speech and language acquisition. This human-specific evolutionary signature underscores the unique role of FOXP2 in the development of human language, distinguishing it from its orthologs in other species.

FOXP2 not only affects language but also involves oral motor coordination, respiratory control, and even mental diseases (such as schizophrenia), which have potential associations (Spiteri et al. 2007). Further elucidation of FOXP2-regulated gene networks and their interactions with neural circuits is needed. As a transcription suppressor, FOXP2 regulates DNA binding ability through heterodimerization or homodimerization and interacts with co-suppressor factors (such as CtBP1) to affect target gene expression. Its regulatory network involves lung and esophageal development, but its functions are particularly prominent in the nervous system, such as regulating T1α, N-myc, and other genes that affect lung development (Li et al. 2004).

The discovery of FOXP2 has opened a new chapter in the study of language genetics, but its role should be understood within a multi-gene and multi-factor framework. FOXP2 plays a central role in language and vocalization learning by regulating neurogenesis, synaptic plasticity, and motor coordination circuits. Its dynamic expression patterns (such as acute downregulation induced by tweets or social interactions) reveal a direct correlation between behavior and gene regulation. Despite the convergent evolution of FOXP2 function in different species, its molecular mechanisms and target gene networks may be species-specific, providing important clues for understanding the biological basis of speech disorders and vocal learning. Furthermore, FOXP2 gene mutations can lead to severe speech and language disorders, such as in the case of the KE family, where FOXP2 heterozygous mutations lead to developmental speech apraxia (DVD), characterized by disarticulation, grammatical comprehension, and language processing deficits (Watkins et al. 2002). In mouse models, Foxp2 biallelic knockout resulted in the complete loss of ultrasonic vocalization (USV) in young mice, while heterozygous mutation resulted in abnormal vocalization patterns, indicating its regulatory role in social communication behavior (Castellucci et al. 2017). Additionally, FOXP2 is highly expressed in areas such as the basal ganglia (especially the striatum), cerebellar Purkinje cells, and thalamus, which are involved in motor coordination, procedural memory, and language processing (Ferland et al. 2003). For example, FOXP2-specific expression in striosomes of the striatum may be associated with the procedural memory of language, and disruption of Foxp2 in mouse models leads to abnormal striosomes and cerebellar development problems, especially damage to Purkinje cells (Chen et al. 2016). Truncated mutations in FOXP2 can lead to developmental speech and language disorders, such as speech apraxia, and FOXP2 may be one of the susceptibility genes for speech disorders (MacDermot et al. 2005).

It is worth noting that while FOXP2 function in vocal learning is conserved across species such as humans, mice, and songbirds, species-specific molecular mechanisms and neural circuit adaptations exist. For instance, although FOXP2 is crucial for song learning in zebra finches and speech development in humans, the specific downstream targets and regulatory networks may vary. Therefore, findings from animal models should be interpreted within their respective species contexts, and extrapolations to human disorders require validation in human-derived systems.

## FOXP2 and ASD, speech disorders

Converging evidence from clinical and preclinical studies underscores the link between FOXP2 dysregulation and the core features of ASD, particularly through its impact on neural circuits involved in language and executive function. Clinical investigations have consistently reported downregulation of FOXP2 expression in individuals with ASD. This reduced expression correlates not only with the severity of language impairments but also with measurable deficits in executive functions and distinct electroencephalographic (EEG) abnormalities, such as decreased α- and γ-wave activity in the prefrontal cortex alongside increased θ-waves in occipital regions (Haghighatfard et al. 2022). These associations suggest that FOXP2 underexpression could serve as a potential biomarker for ASD-related speech disorders (Haghighatfard et al. 2022).

The pathological influence of FOXP2 likely stems from its critical role in the development and function of key brain regions. FOXP2 is highly expressed in the basal ganglia and cerebellum—structures vital for motor coordination, procedural memory, and language processing. In ASD, abnormalities in these regions, especially in cerebellar Purkinje cells, are frequently observed and are thought to contribute to language and social deficits (Fujita et al. 2012, Khouri-Farah et al. 2024). Mechanistically, downregulation of FOXP2 may disrupt the development of corticostriatal and cerebello-thalamo-cortical pathways, impairing synaptic plasticity, neuronal migration, and the fine-tuning of neural networks necessary for fluent speech and social communication (Fujita et al. 2012, Enard 2011). This mechanistic framework is supported by animal models, where Foxp2 mutations lead to cerebellar hypoplasia, Purkinje cell dendritic abnormalities, and deficits in vocalizationand motor learning, phenotypes that parallel core symptoms of ASD (Fujita et al. 2012, Khouri-Farah et al. 2024).

A Neural-Circuit Model of FOXP2-CNTNAP2 Dysfunction in ASD and Speech Disorders.

Converging evidence from clinical, genetic, and animal studies points to a disrupted cortico-striato-thalamo-cerebellar circuit as a central mechanism underlying speech and language impairments in ASD. This model integrates the roles of the basal ganglia, cerebellum, and cerebral cortex in vocal learning, motor planning, and social communication.

•Cortico-Striatal Pathway (Basal Ganglia Loop):

- FOXP2 is highly expressed in the striatum and regulates synaptic plasticity in corticostriatal projections.
- CNTNAP2 supports myelination and neuronal migration in these pathways.
- Dysfunction leads to impaired procedural learning, motor sequencing, and articulation planning—core deficits in speech apraxia and ASD.

•Cerebello-Thalamo-Cortical Pathway (Cerebellar Loop):

- FOXP2 influences Purkinje cell development and cerebellar synaptic integrity.
- Cerebellar output to thalamus and prefrontal cortex modulates motor timing, rhythm, and cognitive-linguistic integration.
- Abnormalities result in atypical prosody, motor incoordination, and impaired social voice processing.

•Integrated Circuit Disruption:

- FOXP2-CNTNAP2 dysregulation disrupts the balance between these parallel loops, leading to:
- Motor Speech Deficits: via basal ganglia and cerebellar motor loops.
- Prosodic and Social Communication Deficits: via cerebellar-thalamo-prefrontal cognitive loops.
- Auditory Processing Abnormalities: via temporal lobe connectivity modulated by CNTNAP2.
- This model provides a parsimonious framework linking gene-level disruptions (FOXP2, CNTNAP2) to circuit-level dysfunction and ultimately to the behavioral phenotypes of ASD and speech disorders.

In summary, FOXP2 dysfunction disrupts a coordinated cortico-striato-cerebellar network essential for speech and language processing. This network integrates:

- Basal ganglia circuits for motor planning and procedural learning,
- Cerebellar circuits for timing, rhythm, and sensorimotor integration,
- Cortical regions (prefrontal, temporal) for social communication and auditory processing.

Downregulation of FOXP2 impairs synaptic plasticity, neuronal migration, and circuit connectivity within this network, leading to the triad of language impairment, executive dysfunction, and altered neural synchrony observed in ASD.

In summary, FOXP2 likely plays a key role in language disorders in ASD by influencing cerebellar development and basal ganglia function. Furthermore, downregulation or dysfunctional FOXP2 may impair neuroplasticity, disrupt synaptic connections, and alter electrical activity. These alterations ultimately contribute to the core language and social communication deficits observed in ASD. Future studies are needed to further elucidate the specific role of FOXP2 in ASD subtypes and explore therapeutic strategies that target and regulate its expression or downstream pathways.

Cerebellar Abnormalities in FOXP2 Mutant Models and ASD Patients.

Abnormal development and function of cerebellar Purkinje cells have been consistently documented in both FOXP2 mutant models and individuals with ASD. In Foxp2 mutant mice, structural deficits include dendritic atrophy, reduced spine density, and diminished expression of synaptic proteins such as synaptophysin, which collectively impair synaptic plasticity and circuit integrity within the cerebellum (Khouri-Farah et al. 2024, Fujita et al. 2008). Functionally, these alterations lead to impaired motor coordination, disrupted vocalization patterns, and deficits in timing and rhythm processing—key components of fluent speech and prosody.

In ASD patients, neuroimaging and postmortem studies similarly reveal cerebellar hypoplasia, altered Purkinje cell morphology, and abnormal functional connectivity within cerebello-thalamo-cortical loops (Varghese et al. 2017, Xiao et al. 2014). These abnormalities are thought to contribute to the motor incoordination, atypical prosody, and impaired sensorimotor integration commonly observed in ASD. Importantly, downregulation of FOXP2 expression correlates with these cerebellar deficits, suggesting a shared mechanistic pathway underlying speech and language impairments in ASD and FOXP2-related disorders.

## FOXP2-CNTNAP2 pathway, ASD, and speech disorder

**Fig. 1:**
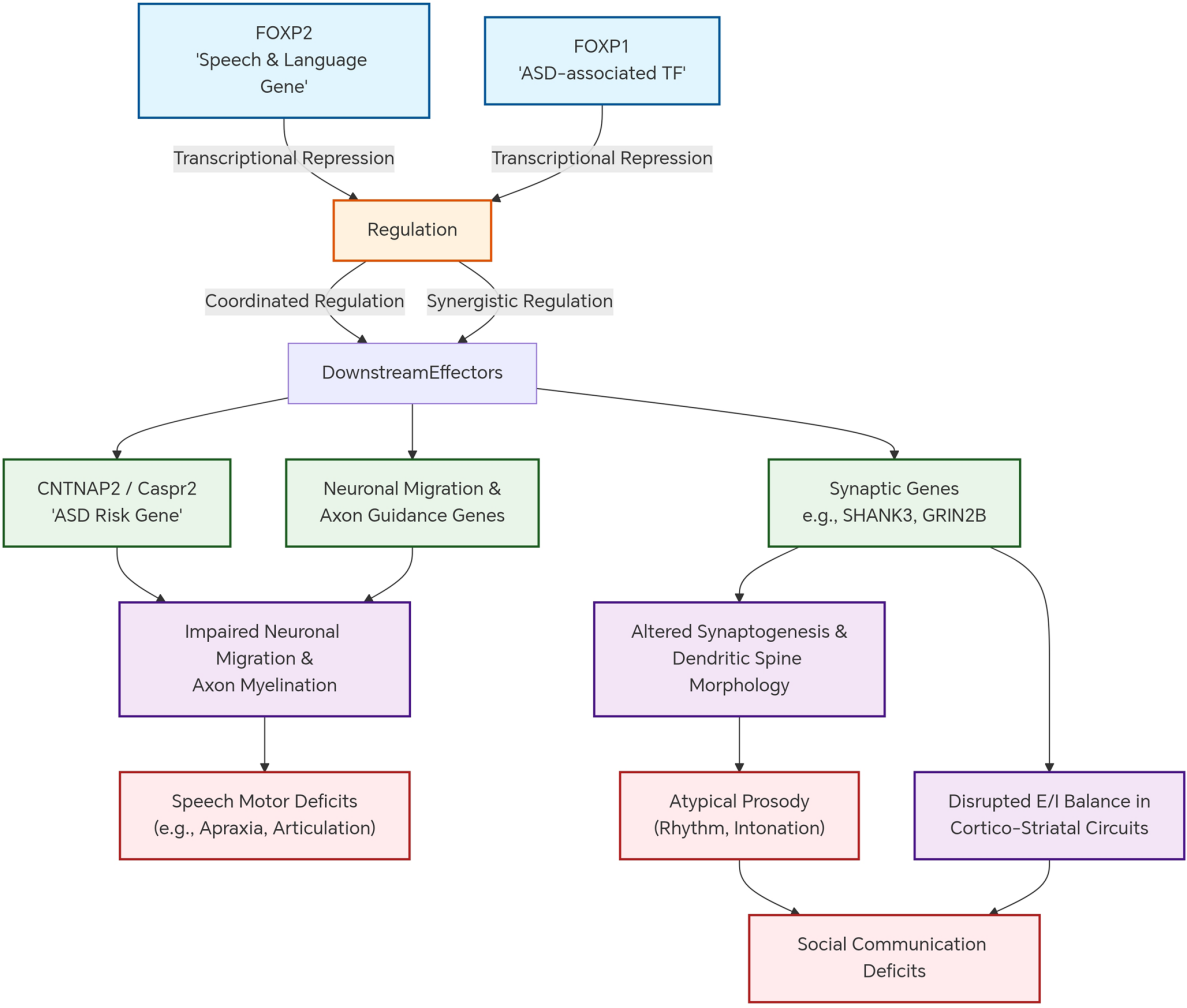
The FOXP2-CNTNAP2-Foxp1 Gene Regulatory Network in ASD and Speech Disorders. Schematic representation of the core gene regulatory network involving FOXP2, CNTNAP2, and Foxp1 in the pathogenesis of Autism Spectrum Disorder (ASD) and speech disorders. FOXP2 (a central speech/language-related transcription factor) directly represses its downstream target CNTNAP2, a major ASD risk gene. Foxp1 functions synergistically with FOXP2 to regulate synaptic genes and neuronal development genes. Dysregulation of this network leads to impaired neuronal migration, altered synaptogenesis, and disrupted excitation/inhibition (E/I) balance in key brain circuits (e.g., cortico-striatal pathways). These molecular and cellular deficits ultimately manifest as the core behavioral symptoms of speech motor impairments, atypical prosody, and social communication deficits characteristic of ASD and related speech disorders. Solid arrows indicate direct transcriptional regulation or primary mechanistic links; the convergence at the circuit and behavioral levels represents integrated pathological outcomes. TF: Transcription Factor.

While FOXP2 has been extensively studied as a central regulator of language-related circuits, its downstream target CNTNAP2 plays an equally critical role in mediating the molecular and neural mechanisms underlying ASD and speech disorders. The detailed exploration of FOXP2 reflects its historical prominence as the first gene linked to inherited speech disorders and its conserved role across species. However, CNTNAP2 serves as a key effector within this pathway, directly influencing neuronal connectivity, myelination, and synaptic function. The interplay within this FOXP2-CNTNAP2-Foxp1 network (Fig. 1) elucidates a common molecular pathway that may underlie the co-occurrence of speech deficits and core ASD symptoms. The following section highlights the specific contributions of CNTNAP2 to ASD pathophysiology, complementing the upstream regulatory role of FOXP2 and providing a more integrated view of the pathway.

CNTNAP2 is a risk gene for ASD. It encodes the Caspr2 protein, which belongs to the neurexin superfamily and functions as a neuronal transmembrane protein involved in cell adhesion, axon myelination, and synaptic organization (Fujita et al. 2012, Peñagarikano and Geschwind 2012). Deletion or mutation of the Caspr2 protein can disrupt these processes, leading to abnormal corticostriatal-thalamic circuits that are crucial for language development and social behavior (Peñagarikano and Geschwind 2012). Consequently, CNTNAP2 variants are associated with all three core symptoms of ASD: social deficits (e.g., reduced interactions), repetitive behaviors (e.g., stereotyped movements), and language impairments (e.g., delayed or degraded language development). This link is strongly supported by animal models, such as Cntnap2-knockout mice, which reproduce these behavioral phenotypes (Fujita et al. 2012, Peñagarikano and Geschwind 2012). Furthermore, the gene’s critical role in neurodevelopment is underscored by findings in an Old Order Amish population, where homozygous mutations in CNTNAP2 cause cortical dysplasia-focal epilepsy syndrome (CDFE), a condition in which patients exhibit language regression and ASD features (Strauss et al. 2006).

Similarly, the MET gene encodes a receptor tyrosine kinase (c-Met) that serves as a signaling hub for processes such as neuronal migration, synaptic plasticity, and immune regulation. Both proteins play crucial roles in neurodevelopment and synaptic connectivity, and their dysregulation has been implicated in ASD pathophysiology. The FOXP2-CNTNAP2 pathway converges on key neural circuits for speech and social behavior:

- Corticostriatal circuits: FOXP2 regulates CNTNAP2 expression in striatal neurons, affecting parvalbumin interneuron function and procedural memory.
- Cerebello-thalamo-cortical loops: Both genes influence cerebellar Purkinje cell development and thalamic relay to the prefrontal cortex, impacting motor timing and cognitive-linguistic integration.
- Temporal-auditory pathways: CNTNAP2 supports myelination in auditory processing regions, modulating speech perception and social voice encoding.

Disruption of these coordinated circuits underlies the overlapping phenotypes of ASD and speech disorders.

Mechanistic Insights from Animal Models: Integrating FOXP2 and CNTNAP2 Evidence.

Animal models provide convergent evidence for the role of the FOXP2-CNTNAP2 pathway in vocal learning and social communication. Foxp2 mutant mice show impaired ultrasonic vocalization, cerebellar hypoplasia, and Purkinje cell abnormalities, paralleling speech apraxia in humans. Similarly, Cntnap2 knockout mice exhibit auditory processing deficits, reduced parvalbumin neuron activity in the striatum, and social abnormalities (Truong et al. 2015, Jang et al. 2023). These models collectively highlight disrupted corticostriatal and cerebello-thalamo-cortical circuits as a common mechanism underlying ASD-related speech deficits.

Mouse models demonstrate that Cntnap2 knockout mice exhibit deficits in auditory processing tasks, highlighting the gene’s key role in language-related phenotypes. In these mice, reduced expression of Parvalbumin (PV) neurons in the striatum may disrupt the homeostasis of motor control and language-related neural circuits. PV neuronal dysfunction is, notably, a common feature of ASD (Truong et al. 2015). Abnormal inhibitory synaptic transmission in the hippocampus of Cntnap2-knockout mice (e.g., decreased inhibition around CA1 pyramidal cells) may lead to an imbalance of neural excitation/inhibition, which is associated with the epileptic comorbidities of ASD and speech disorders (Jang et al. 2023). In addition, the behavioral deficits of CNTNAP2 in mouse models, such as social disturbances and repetitive behaviors, and neuronal migration problems in zebrafish models further indicate the role of this gene in neurodevelopment (Wultz et al. 1990). CNTNAP2 is strongly associated with alterations in brain structural and functional connectivity, particularly within the prefrontal and temporal regions. These connectivity changes are thought to underlie language processing deficits and impaired social cognition in ASD. Specifically, reduced white matter integrity and aberrant neural synchronization in CNTNAP2 mutation carriers may disrupt the integration of auditory, motor, and social information, contributing to the phenotypic overlap between ASD and speech disorders (Xiao et al. 2014).

Mutations in CNTNAP2 also affect language-processing areas of the brain (Shiota et al. 2021). Studies have shown that microdeletions or disruptive mutations in the CNTNAP2 gene, leading to loss of functional protein expression, are found in patients with speech disorders. This indicates that CNTNAP2 haploinsufficiency may be directly related to speech disorders. CNTNAP2 and FOXP2 belong to the same pathway, which may explain their joint role in language function (Petrin et al. 2010). The CNTNAP2 gene is also closely related to auditory processing ability, which may be one of the factors influencing language development (Riva et al. 2018). The synergistic effect of CNTNAP2 and FOXP2 may lead to speech deficits by affecting auditory processing (such as tone discrimination and time series analysis) and neural circuit development (Conti et al. 2020). These findings suggest that CNTNAP2 mutations affect both language and ASD symptoms.

CNTNAP2 is the direct transcriptional target of FOXP2, and FOXP2 itself is related to language disorders and is a known language-related gene. CNTNAP2 mutations can lead to specific language disorders (SLI) and speech apraxia (CAS), which meet the criteria of ASD (Condro and White 2014). Studies have shown that FOXP2 affects language disorders by regulating the CNTNAP2 gene. CNTNAP2 is inhibited by FOXP2 in cortical development, and its polymorphism is associated with language delay in patients with ASD, indicating the co-role of the FOXP2-CNTNAP2 pathway in the two diseases (Vernes et al. 2008).

The MET gene is another ASD risk gene(Mukamel et al. 2011). FOXP2 may play an important role in neurodevelopment by inhibiting the expression of MET, and the abnormal expression of MET in the temporal cortex may be involved in the social communicationdeficits of ASD. The expression pattern of MET in the temporal cortex is complementary to FOXP2, and its abnormal function may lead to defects in neural migration and synaptic connection, further aggravating ASD symptoms (Mukamel et al. 2011). This also illustrates the role of FOXP2 in ASD and provides us with another molecular mechanism link.

Mutations in the CNTNAP2 gene contribute to the core symptoms of ASD (social disturbances, repetitive behaviors) and speech disorders by affecting neural network connectivity, auditory processing, neuronal migration, and synaptic function. The mechanism involves the abnormal function of the FOXP2 pathway, PV neurons, and changes in the corticostriatal circuit. FOXP2 plays an important role in the pathogenesis of ASD by regulating neural stem cell differentiation, synaptic plasticity, cerebellar development, and interaction with other ASD risk genes such as MET. Down-regulated expression or abnormal function of FOXP2 may lead to language disorders, executive function deficits, and brain network abnormalities, becoming potential biomarkers and therapeutic targets for ASD.

## Conclusion

FOXP2 is a central gene in language development, mutations of which lead to speech disorders such as developmental apraxia of speech, implicating abnormalities in the basal ganglia and cerebellum. As its downstream target, CNTNAP2 plays a critical role in both ASD and speech disorders, and the pathway formed by the two genes elucidates common mechanisms across different speech disorder syndromes. The FOXP2–CNTNAP2 pathway serves as a shared molecular hub for speech deficits in ASD and isolated speech disorders by regulating the development of the basal ganglia, cerebellum, and cortex. Studies across species (e.g., mice, songbirds) support the conserved role of FOXP2 in vocal learning, though human-specific mechanisms likely involve additional genetic factors.

The mechanisms of the FOXP2–CNTNAP2 pathway outlined in this review offer tangible directions for clinical translation. From a diagnostic perspective, expression levels of key molecules within this pathway, along with associated neural circuit disruptions, could serve as potential biomarkers for the early identification of at-risk individuals, particularly those with speech delay or atypical prosody. Integrating machine learning-based analysis of prosodic features with genetic and neuroimaging data related to this pathway may enable the development of more precise early screening tools. Therapeutically, targeting downstream effectors of this pathway—through pharmacological modulation, gene-editing strategies in preclinical models, or neuromodulation techniques aimed at restoring cortico-striatal-cerebellar circuit integrity—represents a promising avenue for intervention. Early behavioral interventions focusing on prosodic training and social communication could also be personalized based on an individual’s genetic and neurobiological profile linked to this pathway.

Future studies should further investigate interactions between this pathway and other language-related genes (e.g., FOXP1) and elucidate its spatiotemporally specific regulatory mechanisms during human brain development. Translational research bridging animal models and human clinical trials will be essential to validate the diagnostic utility and therapeutic potential of targeting this pathway, ultimately paving the way toward personalized approaches for ASD and language-related disorders.

## Data Availability

All the generated data and the analysis developed in this study are included in this article.
